# Association between Characteristics of Hospitalized Heart Failure Patients with Their Needs

**DOI:** 10.5539/gjhs.v8n6p95

**Published:** 2015-10-20

**Authors:** Maria Polikandrioti, John Goudevenos, Lampros K. Michalis, Ioannis G. Koutelekos, Elpida Georgiadi, Constantine Karakostas, Moses Elisaf

**Affiliations:** 1 Nursing Department, Technological Institute of Athens, Athens, Greece; 2 Cardiology Department, Ioannina University Hospital, Greece; 3 Nurse Thriasio Hospital, Athens, Greece; 4 Department of Internal Medicine, University of Ioannina, Ioannina, Greece

**Keywords:** heart failure patients, hospitalization, needs of heart failure patients

## Abstract

**Introduction:**

During recent years that life expectancy of heart failure patients has been increased, health professionals put more emphasis on assessing their needs in daily clinical practice. The aim of the present study was to explore the association between characteristics of hospitalized heart failure patients with their needs.

**Methods:**

A sample of 190 hospitalized patients with HF, recruited from public hospitals in Greece, was enrolled in the study. Data were collected by the completion of a questionnaire which included socio-demographic and clinical characteristics and the questionnaire “Needs of hospitalized patients with coronary artery disease” which is consisted of 6 subscales. Statistical methods used were Kruskal wallis-test or Mann-Whitney test and Spearmans’ rho coefficient. Multiple regression analysis was performed in order to evaluate the association between patients’ characteristics and the significance of their needs.

**Results:**

124 (65.3%) of hospitalized heart failure participants were men and 89 (46.8%) of participants were more than 70 years old. 145 (76.3%) had prior experience of hospitalization due to heart failure. The need for support and guidance was statistically significantly associated with the degree of information, (p=<0.001). The need for information from the medical and nursing staff was significantly associated with marital status and degree of information (p=0.001 and p<0.001 respectively). The need for need for being in contact with other patient groups, and ensuring communication with relatives was statistically significantly associated with the professional status and degree of information, (p=0.037 and p=<0.001 respectively). The need for individualized treatment and the need for patient’s personal participation to his/her treatment as well as the need to meet the emotional and physical needs were statistically significant associated with the degree of information, (p=<0.001, p=<0.001 respectively). Lastly, the need to trust the medical and nursing staff was statistically significantly associated with the place of residence and the degree of information, (p=0.023 and p<0.001). These results were confirmed by the multiple linear regression after controlling for potential confounders.

**Conclusions:**

Information seems to be of vital importance when assessing the needs of heart failure patients. Therefore, providing elaborate information should be an integral part of their therapeutic regimen.

## 1. Introduction

Heart failure consists a chronic clinical syndrome that involves various problems in everyday life of patients, mainly attributed to the cognitive and physical impairment that accompanies the disease. Moreover, heart failure implies family, social and economic consequences that exert a negative impact on the outcome of the disease as well as on patients’ quality of life (Polikandrioti et al., 2015; Wever-Pinzon, Drakos, & Fang, 2015; Polikandrioti et al., 2010; Polikandrioti, 2008).

In contemporary times, where the ultimate goal of heart failure patients’ therapy is not solely patients’ survival but also improvement of their quality of life, it is understandable why meeting patients’ needs have come to the forefront of clinical practise (Asadi-Lari, Packham, & Gray, 2003a; Asadi-Lari, Packham, & Gray, 2003b; Wilkinson, & Murray, 1998; Hawe, 1996).

The main concern of the medical and nursing staff at clinical settings is to ensure such an environment that provides fulfillment of their potential needs (needs- orientated approach) (Asadi-Lari, Packham, & Gray, 2003a; Asadi-Lari, Packham, & Gray, 2003b; Wilkinson, & Murray, 1998; Hawe, 1996).

Needs orientated approach is related with many benefits such as stress alleviation, more effective collaboration with health care professionals, satisfaction of provided care and better compliance to the therapeutic regimen. Otherwise, failure to meet patients’ needs is associated with longer hospital stay, increased cost of hospitalization and expenditure on each country’s Health Care System (Polikandrioti & Νtokou, 2011; Wilkinson & Murray, 1998; Hawe, 1996).

It is worth noting that the views of health professionals and patients on their needs may overlap significantly. Usually, health professionals focus on the treatment of biological dimension of the disease whereas patients are more interested on practical or financial issues after hospital discharge (Polikandrioti & Νtokou, 2011; Asadi-Lari, Packham, & Gray, 2003a; Asadi-Lari, Packham, & Gray, 2003b; Wilkinson & Murray, 1998; Hawe, 1996).

The aim of the present study was to explore the association between characteristics of hospitalized heart failure patients (HF) with their needs.

## 2. Material and Methods

### 2.1 Study Design

**Study Population:** The sample size of the present study was 190 hospitalized patients with heart failure in a cardiology department or intensive care unit (ICU) of four public hospitals in Attica.

This particular patient sample was a convenience sample.

The study included all hospitalized patients who met the criteria for entry in the research during January 2015 - March 2015.

The inclusion criteria for the research were: a) a good knowledge/use of the Greek language b) having being hospitalized for at least 3 days and c) being diagnosed with heart failure.

Patients who met the inclusion criteria participated in the study after they had been informed for the purposes of the study and consent for their participation had been taken.

### 2.2 Needs’ Assessment

**Instrument:** Data were collected by the completion of a specially designed questionnaire with the method of interview. Specifically, the questionnaire included:

a) Demographic and clinical characteristics of the sample: Gender, age, marital and educational status, place of residence, number of children, years with heart failure, prior experience of hospitalization due to heart failure and the degree of information,

b) The questionnaire ’Needs of hospitalized patients with coronary artery disease’ which included 39 questions regarding potential needs of patients with coronary artery disease during hospitalization (Polikandrioti et al., 2011).

The 39 questions were grouped according to their content in the following 6 subscales of necessities: a) need for support and guidance, b) need for information from the medical-nursing staff, c) need for being in contact with other patient groups, and ensuring communication with relatives, d) need for individualized treatment and for the patient’s personal participation to his/her treatment e) need to meet the emotional needs (e.g anxiety, fear, loneliness) and the physical needs (such as relaxation, sleep, better conditions during hospitalization), f) need to trust the medical-nursing staff.

Participants had to report how significant each question for them was. The Likert type four-scale was used to answer all questions. The four different scales were represented the following answers: No, Little, Μuch and Too Much.

This questionnaire was constructed a) according to the Kristjandottir (1995) questionnaire, that evaluates the needs of hospitalized children’s parents and had been used in Greece by Kyritsi, Matziou, Perdicaris, and Evagelou (2005) b) after evaluating information from literature review on the needs of hospitalized coronary disease patients, and c) after the researchers conducted informal interviews with patients and health professionals.

The questionnaire ’Needs of hospitalized patients with coronary artery disease’ questionnaire had good reliability and validity in Greek population (Polikandrioti et al., 2011).

More in detail, Cronbach’s a for each sub-scale was as following:

1) Need for support and guidance Cronbach’s a: 0.922
2) Need for information from the medical-nursing staff Cronbach’s a: 0.918
3) Need for being in contact with other patient groups, and ensuring communication with relatives Cronbach’s a: 0.865
4) Need for individualized treatment and for the patients’ personal participation in his/her treatment Cronbach’s a: 0.861
5) Need to meet the emotional needs (eg anxiety, fear, loneliness) and the physical needs (such as relaxation, sleep, better conditions during hospitalization) Cronbach’s a: 0.859.
6) Need to trust the medical-nursing staff: 0.923.

The process of filling out the questionnaires took between 15 and 30 minutes.

The study was approved by the Medical Research Ethics Committee of each hospital and was conducted in accordance to the World Medical Association’s Declaration at Helsinki (1989).

### 2.3 Statistical Analysis

Kolmogorov-Smirnov test was used to assess the normality of continues variables (i.e. patients’ needs etc). Categorical variables are presented as absolute and relative (%) frequencies, while continuous variables following normal distribution are presented as mean ± standard deviation and skewed continuous variables are presented as median (interquartile range). Associations between patients’ characteristics and their needs were tested using the Kruskal wallis-test or Mann-Whitney test. For the association between the length of stay in hospital and the needs, Spearmans’ rho coefficient was calculated.

Finally, multiple regression analysis was performed in order to evaluate the association between patients’ demographic and clinical characteristics (independent variable) and the significance of their needs (dependent variable). In the model, all factors that were found to be significantly associated with the needs were entered as independent variables. The results are presented as β coefficients and 95% confidence interval (95% CI).

All reported P values were based on two-sided hypotheses and compared to a significant level of 5%. All statistical analysis was carried out using SPSS program, version 20 (SPSS Inc, Chicago, Il, USA).

## 3. Results

### 3.1 Baseline Demographics and Clinical Characteristics

The baseline demographic and clinical characteristics of patients with heart failure are presented in Table 1. The majority of patients were men, married, and older than 60 years as well as their disease duration was longer than 1 year. Moreover, almost half of the participants lived in Attica, had low educational status (primary education), and two children, were enough informed regarding their disease and had prior experience of hospitalization due to their current disease.

**Table 1:** Baseline demographic and clinical characteristics of patients with heart failure in Greece (N=190)

Socio-demographics	n (%)
Gender	
*Male*	124 (65.3%)
Age (years)	
*<50*	13 (6.8%)
*51-60*	22 (11.6%)
*61-70*	66 (34.7%)
*>70*	89 (46.8%)
Marital status	
*Married/Living together*	131 (68.9%)
*Single/Divorced/separated/Widowed*	59 (31.1%)
Professional status	
*Civil servant*	14 (7.4%)
*Private employee*	27 (14.2%)
*Free lancer*	16 (8.4%)
*Household*	21 (11.1%)
*Retired*	112 (58.9%)
Educational status	
*Primary education*	112 (58.9%)
*Secondary education*	55 (28.9%)
*University/Master-PhD*	23 (12.1%)
Place of residence	
*Attica*	107 (56.3%)
*Capital city*	32 (16.8%)
*Small town*	32 (16.8%)
*Rural*	19 (10.0%)
Number of children	
*None*	24 (12.6%)
*One*	43 (22.6%)
*Two*	81 (42.6%)
*Three or more*	42 (22.1%)
Years with heart failure	
*<1*	43 (22.6%)
*2-5*	74 (38.9%)
*>5*	73 (38.4%)
Prior experience of hospitalization due to heart failure	
*Yes*	145 (76.3%)
Degree of information	
*Much*	84 (44.2%)
*Enough*	90 (47.4%)
*Little/not at all*	16 (8.4%)
Length of stay in-hospital^§^	5 (4-6)

§ Data are presented as median (interquartile range).

**Patients’ needs**

Table 2 presents the level of significance the patients think of their needs. Patients with heart failure considered all six needs of low significance, once the median scores of each need were close to the lower limit of the needs ranges.

**Table 2:** Descriptive data of the sub-scales assessing the importance of the needs of patients with heart failure in Greece

Patients’ needs (range)	Median (IQR)
*Need for support and guidance (9-36)*	11(9-15)
*Need for information from the medical-nursing staff (8-32)*	9(8-13)
*Need for being in contact with other patient groups, and ensuring communication with relatives (6-24)*	12(6-15)
*Need for individualized treatment and for the patient’s personal participation to his/her treatment (6-24)*	9(6-11)
*Need to meet the emotional needs (eg, anxiety, fear, loneliness) and physical needs (such as relaxation, sleep, better conditions of treatment) (7-28)*	9(7-12)
*Need to trust the medical and nursing staff (2-8)*	2(2-2)

### 3.2 Association of Needs and Demographics

The association between the baseline demographic and clinical characteristics of patients with their needs based on univariate analyses, is presented in Tables 3-5.

**Table 3:** Association between socio-demographic, clinical characteristics of patients with heart failure and 1st-2nd factors of needs in Greece (N=190)

Socio-demographics	*Need for support and guidance*		*Need for information from the medical-nursing staff*	

	Median(IQR)	p-value	Median(IQR)	p-value
Gender		0.989		0.628
*Male*	11(9-15)		9(8-13)	
*Female*	10(9-16)		9(8-14)	
Age (years)		0.549		0.085
*<50*	11(9-15)		9(8-13)	
*51-60*	11(9-15)		12(8-14)	
*61-70*	10.5(9-15)		8(8-13)	
*>70*	11(9-15)		9(8-13)	
Marital status		0.235		**0.001**
*Married/Living together*	10(9-15)		8(8-12)	
*Single/Divorced/separated/Widowed*	12(9-17)		12(8-16)	
Professional status		0.100		0.095
*Civil servant*	17.5(10-18)		13(8-16)	
*Private employee*	13(9-18)		13(8-15)	
*Free lancer*	11(9-13)		10(8-13.5)	
*Household*	9(9-13)		8(8-12)	
*Retired*	11(9-14)		8(8-12)	
Educational status		0.062		0.102
*Primary education*	10(9-13)		8(8-12)	
*Secondary education*	13(9-18)		12(8-14)	
*University/Master-PhD*	12(9-17)		8(8-14)	
Place of residence		0.175		0.203
*Attica*	10(9-14)		8(8-12)	
*Capital city*	11(9-15)		9(8-14)	
*Small town*	11.5(9-16)		11(8-13)	
*Rural*	14(9-18)		11(8-16)	
Number of children		0.402		0.239
*None*	13(10-13.5)		11(8-14.5)	
*One*	12(9-17)		10(8-13)	
*Two*	10(9-15)		8(8-13)	
*Three or more*	10.5(9-15)		8.5(8-13)	
Degree of information		**<0.001**		**<0.001**
*Much*	9(9-13)		8(8-11)	
*Enough*	12(9-17)		10.5(8-14)	
*Little/not at all*	15(13-18)*		12.5(9.5-16)*	
Years with heart failure		0.545		0.447
*<1*	10(9-15)		8(8-13)	
*2-5*	11(9-16)		8.5(8-13)	
*>5*	12(9-15)		10(8-13)	
Prior experience of hospitalization due to heart failure		0.336		0.615
*Yes*	11(9-15)		10(8-13)	
*No*	10(9-15)		8(8-14)	
Length of stay in-hospital^§^	-0.063	0.387	-0.004	0.953

* statistically significantly different score after bonferoni correction

**Table 4:** Association between socio-demographic, clinical characteristics of patients with heart failure and 3rd-4th factors of needs in Greece (N=190)

Socio-demographics	*Need for being in contact with other patient groups, and ensuring communication with relatives*		*Need for individualized treatment and for the patient’s personal participation to his/her treatment,*	
	
	Median(IQR)	p-value	Median(IQR)	p-value
Gender		0.686		0.770
*Male*	11.5(6-15)		9(6-11)	
*Female*	12(6-14)		8(6-12)	
Age (years)		0.883		0.440
*<50*	12(6-15)		9(6-11)	
*51-60*	12(6-15)		9.5(8-12)	
*61-70*	11.5(6-16)		8(6-11)	
*>70*	11(6-15)		8(6-12)	
Marital status		0.295		0.183
*Married/Living together*	11(6-15)		8(6-11)	
*Single/Divorced/separated/Widowed*	12(9-15)		9(7-12)	
Professional status		**0.037**		0.063
*Civil servant*	16(14-18)*		11(10-15)	
*Private employee*	12(7-14)		9(7-12)	
*Free lancer*	11(6-14)		7.5(6-9.5)	
*Household*	11(6-15)		8(6-12)	
*Retired*	11(6-15)		8(6-11)	
Educational status		0.659		0.062
*Primary education*	10.5(6-15)		8(6-10.5)	
*Secondary education*	12(9-15)		10(7-12)	
*University/Master-PhD*	12(6-16)		9(6-12)	
Place of residence		0.101		0.78
*Attica*	11(6-14)		9(6-11)	
*Capital city*	12(8-16)		9.5(6-12)	
*Small town*	14(6.5-15.5)		8.5(6.5-11)	
*Rural*	13(8-17)		9(6-14)	
Number of children		0.856		0.866
*None*	12(9-13.5)		9(8-11)	
*One*	11(6-15)		9(6-12)	
*Two*	10(6-15)		8(6-11)	
*Three or more*	12(6-15)		8(6-12)	
Degree of information		**<0.001**		**<0.001**
*Much*	9(6-14)		7(6-10)	
*Enough*	12(8-15)		9(6-11)	
*Little/not at all*	15(12-17)*		12(9-13)*	
Years with heart failure		0.461		0.226
*<1*	11(6-14)		8(6-12)	
*2-5*	12(6-15)		8.5(6-12)	
*>5*	12(6-15)		9(7-11)	
Prior experience of hospitalization due to heart failure		0.752		0.466
*Yes*	12(6-15)		9(6-11)	
*No*	11(6-15)		8(6-12)	
**Spearman rho**	**p-value**	**Spearman rho**	**p-value**
Length of stay in-hospital^¦^	0.014	0.847	0.002	0.978

* statistically significantly different score after bonferoni correction.

**Table 5:** Association between socio-demographic, clinical characteristics of patients with heart failure and 5th-6th factors of needs in Greece (N=190)

Socio-demographics	*Need to meet the emotional and physical needs*		*Need to trust the medical and nursing staff*	
	
	Median(IQR)	p-value	Median(IQR)	p-value
Gender		0.560		0.514
*Male*	8.5(7-12)		2(2-2)	
*Female*	10(7-12)		2(2-2)	
Age (years)		0.668		0.359
*<50*	9(7-12)		2(2-2)	
*51-60*	10(7-11)		2(2-2)	
*61-70*	8(7-12)		2(2-2)	
*>70*	9(7-12)		2(2-2)	
Marital status		0.054		0.569
*Married/Living together*	8(7-12)		2(2-2)	
*Single/Divorced/separated/Widowed*	10(8-13)		2(2-2)	
Professional status		0.075		0.093
*Civil servant*	12(10-15)		2(2-4)	
*Private employee*	9(7-13)		2(2-4)	
*Free lancer*	8(7-9)		2(2-3)	
*Household*	9(7-12)		2(2-2)	
*Retired*	9(7-12)		2(2-2)	
Educational status		0.196		0.532
*Primary education*	8(7-12)		2(2-2)	
*Secondary education*	11(7-12)		2(2-3)	
*University/Master-PhD*	9(7-13)		2(2-2)	
Place of residence		0.079		**0.023**
*Attica*	8(7-12)		2(2-2)	
*Capital city*	10.5(8-13)		2(2-3)	
*Small town*	10.5(7-12)		2(2-2)	
*Rural*	11(7-15)		2(2-4)*	
Number of children		0.560		0.915
*None*	10.5(7.5-12.5)		2(2-2)	
*One*	10(7-12)		2(2-2)	
*Two*	8(7-12)		2(2-2)	
*Three or more*	9(7-12)		2(2-2)	
Degree of information		**<0.001**		**0.001**
*Much*	7.5(7-11)		2(2-2)	
*Enough*	10(7-13)		2(2-3)	
*Little/not at all*	12(10.5-14.5)*		2(2-4)*	
Years with heart failure		0.699		0.771
*<1*	8(7-12)		2(2-2)	
*2-5*	8(7-12)		2(2-2)	
*>5*	10(7-12)		2(2-2)	
Prior experience of hospitalization due to heart failure		0.963		0.598
*Yes*	9(7-12)		2(2-2)	
*No*	9(7-12)		2(2-2)	
**Spearman rho**	**p-value**	**Spearman rho**	**p-value**
Length of stay in-hospital^¦^	-0.029	0.694	-0.024	0.740

* statistically significantly different score after bonferoni correction.

It was found that the degree of information was significantly associated with the need for support and guidance. In particular, patients with little or not at all information considered this need to be of a much higher significance than patients that were very or enough informed (median 15 vs. 9 and 12, p<0.001) (Table 3).

Marital status, and degree of information were significantly associated with the need for information from the medical-nursing staff (p=0.001, and p<0.001 respectively). More specific, single patients considered this need more significant compared to married patients (median 12 vs 8). Moreover, patients with little or not at all information considered this need to be of a much higher significance than patients that were very or enough informed (median 12.5 vs. 10 and 8) (Table 3).

The need for being in contact with other patient groups, and ensuring communication with relatives, was statistically significantly associated with the professional status, and degree of information (p=0.037 and p<0.001 respectively). In particular, civil servants considered this need to be more important than other patients (median 16). Moreover patients with little or not at all information considered this need to be of a much higher significance than patients that were very or enough informed (Table 4).

The need for individualized treatment and for the patient’s personal participation to his/her treatment as well as the need to meet the emotional and physical needs were statistically significant associated with the degree of information (p<0.001, p<0.001 respectively, Tables 4 and 5).

Lastly, the need to trust the medical and nursing staff was statistically significantly associated with the place of residence and the degree of information (p=0.023 and p<0.001 respectively). Patients living in rural places considered this need to be more significant than other patients. Moreover, patients with little or not at all information considered this need to be of a higher significance than patients that were very or enough informed (Table 5).

### 3.3 Linear Regression Analysis

Linear regression analysis, using the patients characteristics that were statistically significant associated with the needs as possible confounders, revealed that characteristics remain to be statistically significant associated with the needs, was the need to be informed from the medical and nursing staff (see Appendix, Tables 1-3).

## 4. Discussion

The aim of the present study was to explore needs of hospitalized heart failure patients. In clinical environment, fulfillment of patients’ needs is a complicated issue. Usually, patients are reluctant to report their needs whereas health professionals fail to encourage patients to express their needs for various reasons such as lack of leisure time, shortage of nursing staff or the nature of the event (acute or chronic). Additionally, the current trend for early hospital discharge, is minimizing the available time for needs’ evaluation including the need of information (Sepucha et al., 2010; Chevalier, Lombrail, & Gasquet, 2008; Moret, Rochedreux, Chevalier, Lombrail, & Gasquet, 2008; Ivarsson, Larsson, Lührs, & Sjöberg, 2007; Timmins, 2005; Kelly, 2004; Worth, Tierney, & Watson, 2000).

In this research, how well was the patient informed was categorized as following: a) much, b) enough, c) little and d) not at all. The degree of patients’ information was significantly associated with the: 1. need for support and guidance, 2. need for information from the medical-nursing staff, 3. need for being in contact with other patient groups, and ensuring communication with relatives, 4. need for individualized treatment and for the patient’s personal participation to his/her treatment, 5. need to meet the emotional needs (e.g anxiety, fear, loneliness) and the physical needs (such as relaxation, sleep, better conditions during hospitalization), and 6. need to trust the medical-nursing staff.

To the best of our knowledge, there is not a similar research to explore the relation between the degree of information and each need of heart failure hospitalized patients, separately.

For this reason, researchers comment on the significant role of information to cardiac patients which is well documented in the literature. It is also highlighted that information consists a dynamic need that changes over time due to the disease progression. Therefore, significant concerns should be raised about constant reassessment of patients’ level of information (Polikandrioti & Babatsikou, 2013; Polikandrioti & Νtokou, 2011; Johnson & Sandford, 2005; Timmins, 2005; Chan, Reid, Farvolden, Deane, & Bisaillon, 2003).

Relevant research has shown that lack of information is related to distrust of health professionals’ options or uncertainties about the disease. As a consequence, patients experience unwillingness to follow the proposed therapeutic intervention (Polikandrioti & Νtokou, 2011; Sepucha et al., 2010; Moret, Rochedreux, Chevalier, Lombrail, & Gasquet, 2008; Ivarsson, Larsson, Lührs, Sjöberg, 2007; Kelly, 2004; Timmins, 2005; Worth, Tierney, & Watson, 2000).

It is worth noting that information has beneficial effects on the outcome of the disease thus underlining the need for early and appropriate scheduling for providing elaborate information (Polikandrioti & Νtokou, 2011; Sepucha et al., 2010; Moret, Rochedreux, Chevalier, Lombrail, & Gasquet, 2008; Ivarsson, Larsson, Lührs, Sjöberg, 2007; Luttik, Jaarsma, Moser, Sanderman, & van Veldhuisen, 2005; Timmins, 2005; Kelly, 2004; Worth, Tierney, & Watson, 2000).

Interestingly, clinical observation has shown that evaluation of patients’ personality is essential when adopting needs orientated approach and more specifically the need of information. For instance, provision of unnecessary information should be discouraged to stressful personalities since they perceive it as a source of stress. On the other end of the spectrum, provision of detailed information may be beneficial for patients who wish to monitor closely the progress of the disease.

Information to patients with chronic disease, such as heart failure seems to be a vicious circle. In the early stage of the disease, detailed information motivates individuals to seek for behavior changes and gain familiarity with the demands of the disease. Moreover, information allows patients to develop adaptive mechanisms and become able to handle with their needs more effectively. Meanwhile, disease progression, often makes health professionals unable to offer any further medical aid. As a consequence, patients experience alienation and abandonment during a period that their emotional and physical needs are high. In such cases, frequent contact with healthcare professionals provides a frame for their needs’ evaluation.

In terms of marital status, the need to be informed by the medical and nursing staff was significantly associated with single participants. A possible explanation is that being deprived of family support and encouragement, single patients usually turn to health professionals to fulfill their needs. Along with the lack of supportive environment, they may experience difficulties to handle alone with practical issues or performing daily activities. An alternative suggestion is that single heart failure patients are usually unable to express their emotions thus seeking for assistance to medical and nursing staff.

Regarding participants’ profession, civil servants considered the need for being in contact with other patient groups and ensuring communication with relatives to be more important than other patients. Although the relation between profession and patients’ needs has not been fully explored, however it appears that the socio-economic state including the “nature” of the profession may determine up to some extent patients’ needs. Possibly, civil servants, given their professional status are not in a hurry to return to work. However, this finding may reflect the important role of social support.

Interestingly, literature demonstrates the beneficial effect of social support on the outcome of heart failure disease. The prevailing view is that social interaction promotes health because it maintains a rhythm of life (Luttik, Jaarsma, Moser, Sanderman, & van Veldhuisen, 2005; Arthur, 2006; Murberg, & Bru, 2001; Krumholz et al., 1998).

The need to trust the medical and nursing staff was statistically significantly associated with the place of residence (rural areas). Possibly, patients living in rural areas still maintain wide support network. The rapid evolution in human societies, has not yet marked any changes in the nature of rural life including perceptions, values and behavior pattern of individuals. Following this line of thought, it is possible that they wish to develop strong ties of trust and mutual comprehension with health professionals. Significantly more, patients living in rural areas may confront with difficulties of frequent or easy access to health services, thus experiencing this need of high importance.

## 5. Conclusions

Heart failure patients seem to seek for information. Place of residence, occupation and marital status should be evaluated when assessing needs of hospitalized heart failure patients’.

Though, needs orientated approach may demand better organization of health services, however it provides high quality of care and improvement of the therapeutic effect, thus reducing hospitalization costs.

## 6. Limitations of the Study

The study sample was not representative of patients with heart failure in Greece, but a convenience sample. The relevant sampling method limits the ability generalization of results.

Furthermore, since this study is a sub-analysis of a bigger one that was conducted the previous years, having different endpoints, power analysis for this study was not performed. (Polikandrioti, 2012)

## Appendix

**Table 1:** Multiple regression for the 1st-2nd need of patients with heart failure

Socio-demographics	*Need for support and guidance*		*Need for information from the medical-nursing staff*	
	
	β coefficient	p-value	β coefficient	p-value
Marital status					
*Married/Living together*	-			Ref. Cat.	
*Single/Divorced/separated/Widowed*	-			2.19(1.10-3.28)	**<0.001**
Degree of information					
*Much*	Ref. Cat.			Ref. Cat.	
*Enough*	1.83(0.58-3.09)	**0.004**		1.96(0.92-3.01)	**<0.001**
*Little/not at all*	3.96(1.69-6.23)	**0.001**		3.48(1.58-5.38)	**<0.001**

**Table 2:** Multiple regression for the 3rd-4th need of patients with heart failure

Socio-demographics	*Need for being in contact with other patient groups, and ensuring communication with relatives*		*Need for* individualized treatment and for the patient’s personal participation to his/her treatment	
	
β coefficient	p-value	β coefficient	p-value
Professional status					
*Civil servant*	Ref. Cat.			-	
*Private employee*	-3.55(-6.27- -0.84)	**0.011**		-	
*Free lancer*	-3.59(-6.62- -0.57)	**0.020**		-	
*Household*	-3.75(-6.60- -0.91)	**0.010**		-	
*Retired*	-3.72(-6.05- -1.41)	**0.002**		-	
Degree of information					
*Much*	Ref. Cat.			Ref. Cat.	
*Enough*	1.88(0.64-3.12)	**0.003**		1.41(0.40-2.40)	**0.006**
*Little/not at all*	4.17(1.92-6.42)	**<0.001**		2.97(1.16-4.78)	**0.001**

**Table 3:** Multiple regression for the 1st-2nd need of patients with heart failure

Socio-demographics	*Need to meet the emotional and physical needs*		*Need to trust the medical and nursing staff*	
	
	β coefficient	p-value	β coefficient	p-value
Place of residence				
*Attica*	-	Ref. Cat.	
*Capital city*	-	0.20(-0.11-0.51)	0.201
*Small town*	-	0.05(-0.25-0.36)	0.731
Rural	-	0.70(0.32-1.08)	**<0.001**
Degree of information				
*Much*	Ref. Cat.		Ref. Cat.	
*Enough*	1.61(0.63-2.59)	**0.001**	0.38(0.15-0.61)	**0.001**
*Little/not at all*	3.22(1.46-5.01)	**<.001**	0.65(0.23-1.07)	**0.003**
